# Health Week and Its Objects: A Story of Progress

**Published:** 1924-03

**Authors:** 


### HEALTH WEEK AND ITS OBJECTS.

### A STORY OF PROGRESS.

The object of Health Week is to bring home to every man, woman and child in the country a sense of the importance of health, and their personal responsibility for safeguarding it. This great object has been left too much to the specialist—the doctor, the sanitary inspector and the engineer. We forget that these officers, zealous and hardworking as they are, can cover only a part of the ground. No medical officer, however capable, can do a quarter as much to make or mar the health of a family as the woman in the home. Health Week seeks to focus public attention for one week in the year on matters of health, and to arouse that sense of personal responsibility without which all Public Health activities must fall far short of their aim. It endeavours in the first place to enlist support for the work of our Public Health authorities, and secondly, to show people how much they can do for themselves.

### The Origins.

Health Week sprang from a suggestion dropped early in 1911, in a presidential address to the Institution of Sanitary Engineers. The idea was warmly received by the press, and later in the same year a strong Committee of the Agenda Club was formed to carry it into effect. Under their guidance local committees were established throughout the country, and in April and May, 1912, the first Health Week celebration took place. Full programmes, covering the whole week, were carried through in 41 towns. Less complete celebrations, including sermons, lectures, public meetings, and lessons in the Sunday-schools and day-schools, took place in some hundreds of towns and villages. A distinct advance was made in 1913, the great manufacturing centres being conspicuous for the excellence of their programmes and the energy with which they were carried through.

### The War and Baby Week.

The value of Health Week was so clearly demonstrated by these two celebrations that it was felt that the institution should be placed on a permanent footing. Accordingly in 1914, at the request of a meeting of local authorities, the Royal Sanitary Institute, our premier health society, which embraces doctors, engineers, architects, sanitary inspectors, nurses, health visitors, and every other health-promoting profession, appointed a Committee to take charge of the Central Organisation. Then came the War; and the Central Committee, feeling that every effort should be concentrated on that one supreme issue, suspended their operations till 1920. Meanwhile, the appalling toll of the nation's manhood taken by the War had led to the institution of National Baby Week, with the object of focusing attention on all that has to do with Infant Welfare. The aims of the two movements are very similar, and the central committees responsible for them are working in the closest co-operation. Baby Week is held in July; the Health Week Committee find it easier to get people together in the autumn. Last year's Health Week was celebrated from October 8th to 14th, in some 100 centres in Great Britain and the Empire.

### The Force of Public Opinion.

Health Week is essentially a war on ignorance. It is safe to say that more people die from ignorance of the laws of health than from any other single cause. Health Week helps to raise the standard of living. Many people are poor for want of money, but many more for want of knowledge. Many a family lives better on £2 a week than others do on £10. The force upon which Health Week relies is public opinion. It seeks to stir up a healthy local rivalry between town and town and village and village, and to build up a public opinion which will be ashamed of a high disease rate, or an excessive infant mortality, and will feel as a communal reproach the sight of an ill-nourished or decrepit child. Health Week embraces every agency which seeks in any way to promote the well-being of the people. The clergy can awaken and stir the consciences of their congregations, and bring home to them a sense of personal responsibility for their own health and that of those dependent on them. The Local Authorities are doing a great work, the value of which is rarely appreciated. In Health Week they have an opportunity of taking their constituents into their confidence and telling them of what has already been accomplished, and of that which still remains to be done.

### "Many Inventions."

The thousands of essays which have been received year after year from the London County Council Schools show how well the children have profited by their instruction. Children can be reached through the eye as well as the ear. Ten thousand school-children at Bootle attended lantern lectures on health in 1922. At Hull last autumn two thousand children listened with breathless interest to a similar lecture. Valuable help has also been received from co-operative and friendly societies, Guilds of Help, and Women's Institutes—every body of men and women, in short, who care for the well-being of their fellow-citizens, and are willing to work for it. The observance of the week has necessarily been left to the discretion of the Local Committees. These Committees have vied with one another in devising new and ingenious ways of reaching the people. Exhibitions, healthy houses, films, demonstrations in cookery and first aid, Morris dancing and gymnastic displays, have all been pressed into service.

### Beginning at the Beginning.

To try to instruct the masses of the people in hygiene without first making a serious effort to arrest their attention is to attempt the impossible. But, we are told, people cannot be got to attend lectures and meetings. It all depends on how they are organised. In the East End of London crowded audiences gathered night after night. At Halifax, at the end of a crowded week, two thousand people went to the closing meeting. In quite small towns audiences of five hundred and over have been common. It is ignorance which causes men and women to fall an easy prey to the faddist, the scaremonger and the quack.

